# Patterns of object play behaviour and its functional implications in free-flying common ravens

**DOI:** 10.1038/s41598-024-83856-9

**Published:** 2025-01-02

**Authors:** Awani Bapat, Anna E. Kempf, Salomé Friry, Palmyre H. Boucherie, Thomas Bugnyar

**Affiliations:** 1 Department of Behavioral and Cognitive Biology, University of Vienna, Djerassiplatz 1, 1030 Vienna, Austria; 2 Konrad Lorenz Research Center for Behavior and Cognition, Grünau im Almtal, Core facility of the University of Vienna, Fischerau 13, 4645 Grünau im Almtal, Austria; 3 Department of Evolutionary Biology and Environmental Studies, University of Zurich, Winterthurerstrasse 190, 8057 Zurich, Switzerland

**Keywords:** Behavioural ecology, Social evolution, Animal behaviour

## Abstract

Object play has been proposed to provide individuals with information about their environment, facilitating foraging skills and tool use. In species where object play co-occurs with locomotor or social play, it may have additional functional implications, such as facilitating the evaluation of peers or forming social bonds. For instance, ravens judge others’ competitiveness via play caching and engage in social play by exchanging objects. However, most raven studies were conducted on a restricted number of individuals and under controlled captive settings. To validate these findings and to gauge the functional scope of object play under field conditions, we investigated the patterns and characteristics of object play in individually marked free-flying ravens in the Austrian Alps. Using two large data sets, we show a decrease in object play with age and a preference for playing longer with novel objects, supporting findings from captivity. We also find adults occasionally playing with objects and no difference in the duration of social or non-social object play bouts across age classes. Taken together, our findings are in line with the assumption that raven object play likely has more than one function and serves to gain information about the individual’s physical and social environment.

**Supplementary Information:**

The online version contains supplementary material available at 10.1038/s41598-024-83856-9.

## Introduction

Object play behaviour is characterised by physical interactions with an object by picking it up or combining it with other substrates or objects^1^, typically providing no immediate benefits to the individual engaged in the behaviour^2^. The functions of object play have been attributed mainly to environmental exploration and preparation for uncertainties, which may facilitate the development of foraging skills or tool use required in later life^3–6^. In many species, object play does not occur independently of other play behaviours but merges with social or locomotor play^2^. The functional implications of object play in such cases may go beyond environmental exploration or preparation for uncertainties.

Corvids are renowned for their wide range of play behaviours^7^ and common ravens (*Corvus corax*) have been reported to frequently combine different types of play, such as hanging upside down with object in beak, carrying an object in the foot, and tug-of-war^8,9^. The attraction to objects decreases within the first two years of life^10^, suggesting that a primary function of object play may be environmental exploration. However, ravens may also use object caching to evaluate peers^11^and share/exchange objects with conspecifics^12^, implying that object play may also serve a social function. Moreover, object play is contagious, triggering play in others^13,14^.

Most play studies in corvids have been conducted in captivity, with a restricted number of individuals and under controlled access to objects (e.g^11,15–18^). However, animals in captivity may be more likely to exhibit play behaviours as they experience less stressful conditions regarding foraging or predator avoidance^19^. Furthermore, wild raven groups can be socially highly dynamic^20^: on one hand, group size and composition may change several times during the day^21^, depending on the context (roosting^22^, foraging^23,24^, or socialising^21^) and season^25^; on the other hand, individual birds may develop preferences for certain foraging and socialising sites^26^, resulting in high encounter rates between particular birds at these sites and the formation of dominance rank hierarchies and social bonds^27,28^. Given the combination of moderate to high degrees of fission-fusion dynamics and individual site preferences, non-breeding wild ravens are typically surrounded by individuals with different degrees of familiarity, which stands in sharp contrast to the socially stable conditions in captivity. Thus, the play patterns reported from captive ravens may be specific to context^29^and differ in various aspects from those of wild populations. Nevertheless, it could also be that the intrinsic motivation to engage in object play is similar for captive and wild ravens, and thus the findings from captivity could be validated in the field. Hence, complementing captive studies with those on free-living populations are needed to understand the extent and functions of play (see for instance^30^).

We here examine two data sets on object play in a free-flying population of individually marked ravens that has been monitored for 15 years, to validate findings from captivity and to test predictions derived from the functional hypotheses. If the primary function of object play is to promote environment exploration, we would expect (i) young ravens to play more than adults, (ii) that most of the play involves solitary object manipulation, and (iii) that novel or unfamiliar objects are played with more. However, if the functionality of object play is also associated with the development of social skills and/or relationships, then we would expect (i) that adults also engage in object play and (ii) that the behaviour is also associated with social interactions. Using our first data set collected over nine years, we describe the patterns of occurrence of object play across sex, age class and season, with the aims of validating findings from captivity^10,11,13–16^ and examine whether the object play patterns across age class support the environmental exploration and/or the social skills hypothesis (i). Our second data set is aimed at identifying whether object play behaviour occurs in social or non-social contexts, allowing us to determine support for the social skills hypothesis (ii), and whether the duration of play bouts varies depending on the types of objects chosen for play, providing further evidence for the environmental exploration hypothesis (iii).

## Methods

### Study population

The study has been carried out in the Northen Austrian Alps region of Austria, specifically at the Cumberland Wildpark, Gruenau im Almtal, Upper Austria where a free-flying population of common ravens regularly scavenges the food from the zoo animals’ enclosures^27^. Since 2008, about 600 ravens have been individually marked with a unique combination of colour rings, coloured patagial wing tags, and a numbered metal ring from Vogelwarte Radolfzell (2008–2017) or Austrian Ornithological Centre (2017–2022). Sex was determined genetically from 50 to 200 µl blood taken from the ulnar vein. The age class of individuals was determined based on mouth and feather colouration^31^. Depending on the season, foraging groups consisted of 20–120 ravens, of which on average 51.75% were individually marked. All ravens were habituated to the regular presence of researchers in the wild park.

### Data collection

#### Dataset 1

As part of the long-term monitoring program, focal observational sampling^32^of marked individuals was carried out between 2008–2010, 2013–2015 and 2020–2022. Between 2008–2010 and 2013–2015, the data was collected using digital voice recorders by three separate observers. The sampling was conducted in the morning (7:00–11:00) and afternoon (14:00–18:00) and spread out across the main locations in the wild park used by the ravens^27,33^. For each focal bird, the observers recorded all occurrences and durations of behaviours according to a pre-defined ethogram, including behaviours involving object play: manipulation, transport, caching, co-manipulation, offer/show to conspecific, or transfer to conspecific (Supplementary Table S6). Observers were trained on the ethogram by T.B. but they were not directly compared for inter-observer reliability; we thus controlled for the observer identity as a random effect term in the statistical analysis (see below). Between 2020–2022, the focal data was recorded by 10 observers using hand-held video cameras (Panasonic HC-V700 series) following the sampling protocol described above. The videos of each focal bird were later coded by five coders using the behaviour coding platform Loopy (LoopBio: http://loopbio.com/loopy/). To ensure inter-coder reliability, all coders were trained on a subset of videos, coded by A.B., who served as a reference for all others. All coders reached 85% concordance with the reference coding before starting the main coding. Since inter observer reliability was done between the coders, but not with the observers for data collected between 2008–2010 and 2013–2015, we included all coders as “LoopyGroup” while controlling for observer identity in the statistical analyses (see below).

#### Dataset 2

To further investigate characteristics of object play behaviour, A.B. collected data between October 2021 – December 2023. As described above, the sampling was conducted in the morning and afternoon over all locations in the wild park used by the ravens (mean duration of observation rounds 72.5 min, range 10–150 min). Object play behaviours were sampled opportunistically every time a marked individual was observed interacting with an object (start of play bout) and continued till either when the focal individual stopped physically interacting with it for at least four seconds, or when the focal individual transferred the object to another individual (end of play bout). When a bout of object play was identified, the observer recorded the identity of the individual, the length of the play bout, the behaviours occurring during the play bout, and the type of object they played with, using a digital voice recorder. We classified each play bout type as social or non-social based on whether or not social interactions involving the object (request, offer, transfer, steal, co-manipulate, pilfer) occurred (Supplementary Table S6). We classified the objects into four categories based on their origin: anthropogenic, animal-derived, plant-derived, or natural-inorganic (Supplementary Table S2).

### Statistical analyses

All statistical analyses were carried out in R (version 4.3.2)^34^on a Windows 11 Pro, 64-bit system with an Intel i7 Processor. We fit Generalized Linear Mixed Models (GLMMs)^35^with the package lme4 (version 1.1–35.1)^36^. For model diagnostics and computing confidence intervals, we used functions from the package car (version 3.1-2)^37^ and custom functions kindly provided by Roger Mundry.

#### Model 1

To investigate the patterns of occurrence of object play, we fitted a GLMM with a binomial error structure and logit link function (function glmer of package lme4)^38^. The response was occurrence of object play during the focal protocol (0 = no occurrence, 1 = occurrence, dataset 1). We included sex, age class, and seasons as exploratory predictors. Although we were also interested in the interaction effect of age class and season, we did not include this as a fixed effect predictor as we were missing values for juveniles in spring. This is due to juvenile ravens being trapped and marked only from summer onwards once they have fledged. We included the random effects of focal individual identity, observer identity, and date. We also added all theoretically identifiable random slopes, specifically those of season within focal individual identity, and sex, age class, and season within observer identity. We included focal observation duration as an offset term in the model to control for variation in focal durations. All fixed effects were dummy-coded and centred before being included as random slope terms in the model formula.

#### Model 2

To further investigate the characteristics of object play, we fitted a linear mixed model (LMM, function lmer of package lme4) with the duration of the object play bout as response variable (dataset 2). To examine whether individuals of different age-classes engage in different durations of non-social or social object play, we included the interaction between age class and play type (social or non-social), along with object type, as test predictors. As control predictors, we included sex, age class and season. We also included the random effects of focal individual identity and date to avoid pseudo replication. Before fitting the model, we found the response to have a skewed distribution and hence we log-transformed it to ensure convergence.

For model diagnostics, we visually examined the normal distribution of the best linear unbiased predictors and the normality and homogeneity of the residuals (Supplementary Fig. S2 and S4). We tested for collinearity of the fixed predictors using variance inflation factors (VIF, function vif of package car) which revealed no collinearity issue (model 1: max VIF = 1.037, model 2: max VIF = 1.274). Examination of model stability by excluding each level of the random effect at a time^39^ and comparing the estimates derived from these models to that of the full model revealed good stability for model 1 (Supplementary Fig. S3). However, for model 2, we found that the estimates for season summer, age class Adult, and interaction term of age class Adult and play type social were unstable (Supplementary Fig. S5). Hence, caution must be taken when interpreting the model results, especially regarding these estimates. This is also indicated by the large 95% confidence intervals for these estimates (Table 1), which were computed as described below.

We tested the effects of individual fixed effects with a likelihood-ratio test by excluding each fixed effect term at a time using drop1 function. The confidence intervals of the model estimates were computed using a parametric bootstrap (with 1000 bootstraps, function bootMer of package lme4). Post-hoc pairwise comparisons were carried out by releveling the factor levels and refitting the models.

### Data visualisation

All plots were created using R package ggplot2 (version 3.5.0)^40^. All figures corresponding to the statistical models were generated using R package ggeffects (version 1.7.0)^41^.

### Ethics

All procedures concerning trapping, blood-sampling, marking and behavioural studies of wild ravens were carried out in accordance with Austrian ethical standards, with licenses from the Commission for Animal Experimentation of the Austrian Government (BMWF-66.006/0010–11/10b/2009, BMWF-66.006/0009-II/3b/2012, BMBWF-66.006/0015-V/3b/2018). The monitoring and ringing programme of the Konrad Lorenz Research Centre is authorized by the Central administration of Upper Austria. The studies themselves were purely observational and non-invasive, and thus not classified as animal experiments in accordance with the Austrian law (Federal Law Gazette no. 114/2012, article 1, § 2). All methods are reported in accordance with the ARRIVE guidelines.

## Results

### Dataset 1: Patterns of occurrence of object play across sex, age class, season

**Fig. 1:**
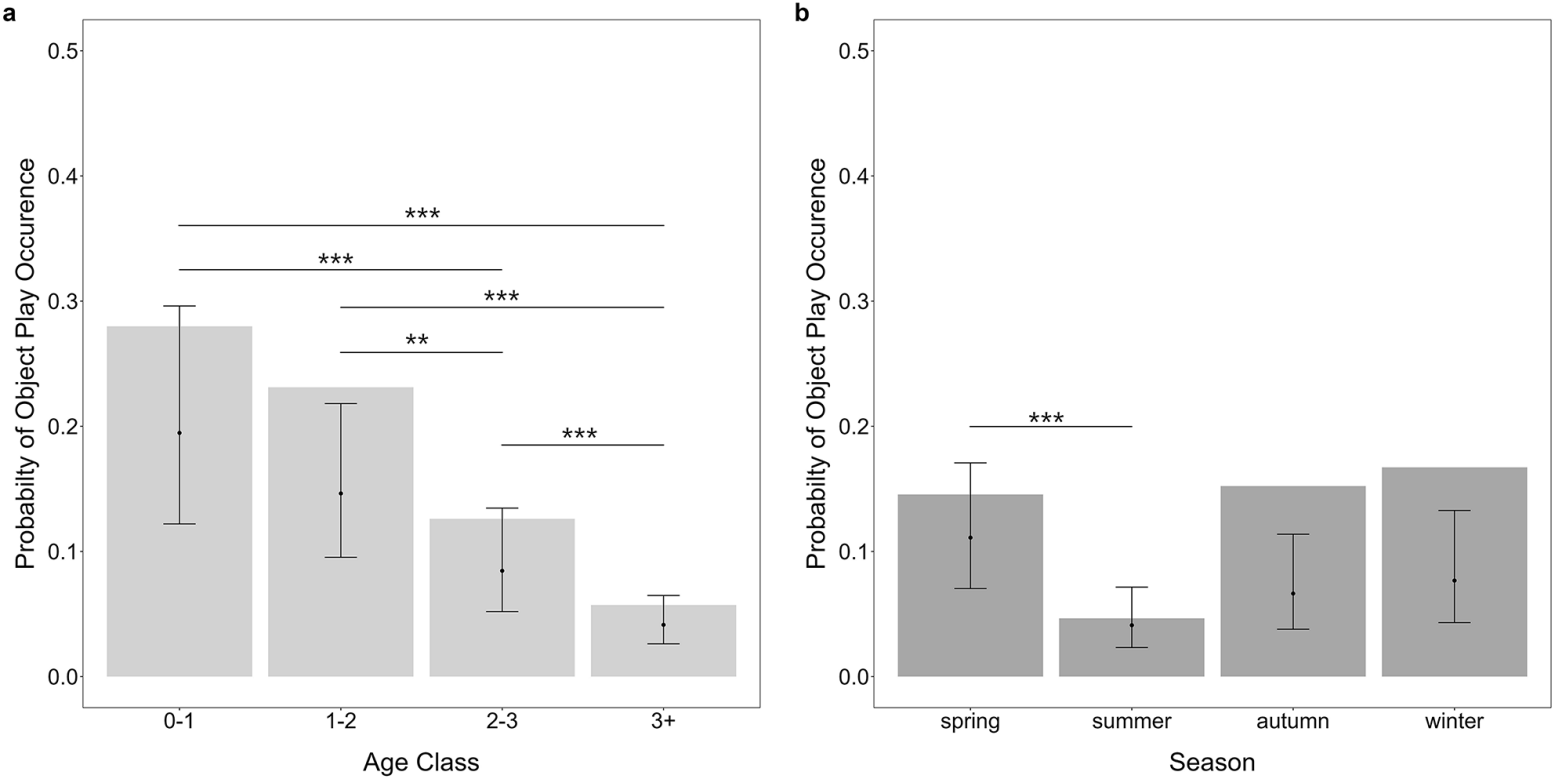
Probability of occurrence of object play across (**a**) age class (0–1: juveniles, 1–2: sub-adult year 1, 2–3: sub-adults year 2, 3+: adults) and (**b**) seasons (spring: March-May, summer: June-August, autumn: September-November, winter: December-February). Bar plots indicate observed average occurrence of object play. Error bars indicate 95% confidence intervals around the estimated marginal means, generated using ggeffects function of R package ggeffects. Significance codes: * < 0.05, ** < 0.01, *** <0.001.

We analysed the patterns of occurrence of object play behaviour from 3257 focal observations (mean duration 3.4 min, range 1–15 min) on 163 individually marked ravens (82 females, 81 males) collected between 2008–2022. Overall, object play occurred in 12% of the focal observations, with 71% of individuals engaging in object play at least once (juveniles: 67%, sub-adults year 1: 83%, sub-adults year 2: 65%, adults: 51%; for details see Supplementary Note S1, Table S1). The comparison between the full and the null model (only including the random effects terms) confirmed that the included predictors meaningfully contribute to explaining the variability in the response variable (GLMM: *χ*^2^ = 26.422, df = 7, *p* = 0.0004). In detail, sex did not significantly explain the occurrence of object play (Table 1: Model 1). However, the probability of occurrence of object play decreased across age classes and varied across seasons (Fig. 1; Table 1: Model 1).

### Dataset 2: Characteristics of object play

**Fig. 2:**
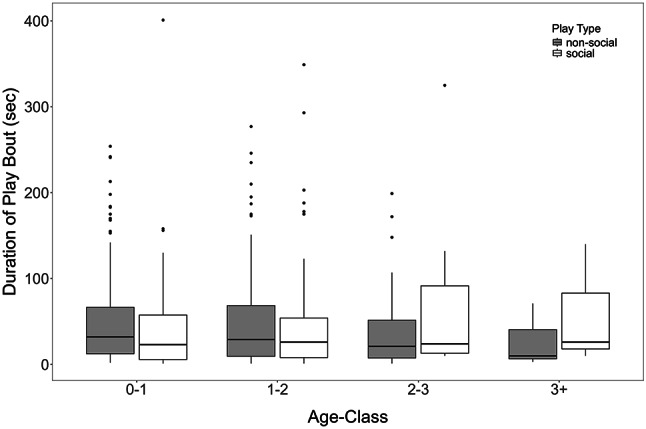
Duration of object play bouts for different play types (non-social or social) across age class (0–1: juveniles, 1–2: sub-adults year 1, 2–3: sub-adults year 2, 3+: adults). Here, we do not show the model outputs as the estimates associated with these predictors were not stable (Supplementary Fig. S5).

**Fig. 3:**
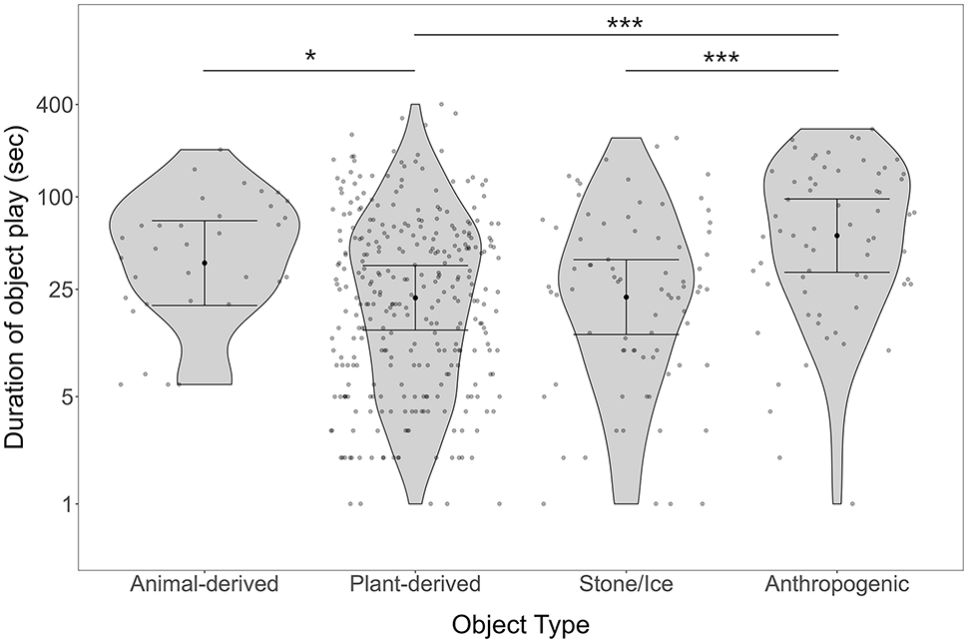
Duration of object play bouts across different object types (animal-derived: bones, feather, fur; plant-derived: leaves, sticks, bark, nutshells, etc.; stone/ice; anthropogenic: plastic, cloth, paper, etc.). Violin and scatter plots indicate distribution of the data within each category of object type. Error bars indicate 95% confidence intervals around the estimated marginal means, generated using ggeffects function of R package ggeffects. Significance codes: * < 0.05, ** < 0.01, *** <0.001.

Between October 2021 – December 2023, we recorded 488 play bouts from 35 individually marked ravens (17 females, 18 males; Supplementary Note S2). To examine the factors explaining the durations of the object play bouts, we compared the full model to a null model (including only fixed effect terms of age class and season, and all random effect terms) and found that it was significant (LMM: *χ*^2^ = 33.843, df = 7, *p* = 0.00002), indicating that the included predictors meaningfully explain the variability in the response. Looking at each test predictor individually, we find no effect of the interaction between age class and play type on the duration of object play (Table 1: Model 2, but see Supplementary Fig. S5). Individuals of all age classes engaged in similar durations of non-social and social play (Fig. 2). Nevertheless, it is interesting to note that 116 out of 270 of the bouts with object manipulations and 14 out of 88 bouts with object caching involved social interactions over the objects, and 27 out of all the 488 bouts involved locomotor play (Supplementary Table S5). Further, object type significantly affected the duration of object play bout (Fig. 3; Table 1: Model 2), irrespective of the age class. While noting descriptively that plant-derived objects were played with most often (311 of 488 play bouts; Supplementary Table S3), we found that individuals played longer with animal-derived or anthropogenic object types (Fig. 3).

**Table 1:** Model results for occurrence of object play (Model 1) and duration of object play bouts (Model 2).

Term	Estimate	SE	95% CL_lower_	95% CL_upper_	*χ* ^2^	df	*p*-value
**Model 1: Occurrence of Object Play (binomial GLMM)**							
(Intercept)	−2.076	0.360	−2.784	−1.349			
Sex male	0.125	0.161	−0.202	0.445	0.576	1	0.448
Age-class Sub-adult Year 1	−0.344	0.240	−0.844	0.096	18.632	3	< 0.001***
Age-class Sub-adult Year 2	−0.963	0.283	−1.561	−0.465			
Age-class Adult	−1.723	0.264	−2.212	−1.196			
Season - Summer	−1.071	0.298	−1.639	−0.522	9.145	3	0.027*
Season - Autumn	−0.563	0.322	−1.208	0.026			
Season - Winter	−0.407	0.320	−1.062	0.217			
**Model 2: Duration of Object Play Bouts (LMM)**							
(Intercept)	3.613	0.324	2.984	4.259			
Sex male	0.226	0.130	−0.023	0.488	3.015	1	0.082
Object-type Anthropogenic	0.411	0.292	−0.193	0.934	28.314	3	< 0.001***
Object-type Natural-Inorganic	−0.511	0.279	−1.081	0.008			
Object-type Plant-derived	−0.522	−0.249	−1.007	−0.074			
Season - Summer	0.241	0.353	−0.399	0.924	4.238	3	0.237
Season - Autumn	0.088	0.231	−0.346	0.551			
Season - Winter	−0.273	0.240	−0.726	0.212			
Age-class Sub-adult Year 1	−0.130	−0.205	−0.535	0.267	5.551	3	0.136
Age-class Sub-adult Year 2	−0.356	0.278	−0.926	0.234			
Age-class Adult	−0.751	0.728	−2.307	0.649			
Play-type Social	−0.156	0.205	−0.546	0.255			
Age-class Sub-adult Year 1: Play-type social	0.062	0.292	−0.508	0.639			
Age-class Sub-adult Year 2: Play-type social	0.708	0.432	−0.184	1.596			
Age-class Adult: Play-type social	1.821	1.050	−0.334	3.872			

Significance codes: * < 0.05, ** < 0.01, *** <0.001. Reference categories are “female” for Sex, “Juvenile” for Age class, “Spring” for Season, “Animal-derived” for Object-type, and “Non-social” for Play-type. Indicated *χ*^2^, df, and p-values refer to the overall effects of the predictors. All estimates for Model 2 are with reference to log-transformed response variable i.e. natural log of duration of object play bout.

## Discussion

We here present the first long-term study on common ravens describing the patterns and characteristics of object play behaviour in a free-flying population. By analysing data collected over 9 years, we find that the occurrence of object play is affected by the ravens’ age class and by season but not by the birds’ sex. Aside from the seasonal effect (that birds play less in summer), the results fit well to those obtained from captive studies, where object play decreased from the juvenile to the subadult period^10^, but no indications of sex differences were found^11,13,15,42^. These patterns also corroborate the main trends in animal play: age effects are common and sex effects tend to be rare in object play^43^; the latter stands in contrast to other forms of play, as sex differences are frequently found in social and locomotor play^2^. While these general patterns are consistent with the assumption of object play facilitating information gain about the physical environment, it is interesting to note that we found (i) free-flying ravens to show object play at similar rates across most of the year (except in summer) and (ii) adults to also engage in object play. The fact that ravens showed high levels of object play across autumn, winter, and spring indicates that this behaviour occurs over periods of varying social and foraging dynamics^21,25,27,28^. The lower object play rates during summer could be a consequence of changes in the social dynamics as the juveniles begin integrating into the non-breeder groups during these months (June-August) and are likely to face more aggression^28^, but needs detailed investigation in future studies. Alternatively, weather conditions may also affect play rates^44,45^, however, to examine this, finer scale data on variations in weather conditions across the seasons is needed. That adult ravens are regularly involved in object play, although in lower numbers than immature birds, further supports the relevance of studying object play under field conditions. However, based on the long-term data, we cannot distinguish whether adults, from time to time, encounter novel objects that they have not yet explored, or whether they sometimes use familiar objects to initiate social interactions with other ravens.

With our second, play-focused data set, we thus investigated whether individuals of different age classes engage in different durations of social or non-social object play. Non-social play included object manipulations or caching, which may or may not be accompanied by locomotor play (lying on the side or rolling down slopes); while social play involved manipulations or caching accompanied by the exchange of objects or co-manipulations, which again may or may not involve locomotor play. We do not find a significant difference in the duration of social or non-social object play bouts across age classes, indicating that young and old ravens partake in either type of object play. This pattern was expected if object play serves either function, to gain information about the physical and the social environment. How quickly object play can become a social event may be illustrated by the fact that about one third of the play bouts involving object manipulations resulted in interactions over the object whereas, 13% of the play bouts involving caching resulted in interactions over the cached items (Supplementary Table S5). Examining whether contagious play bouts tend to be of longer durations than non-contagious bouts, as found in a recent study^46^, may be more informative in the future with regards to the functional role of object play in the development of social relationships. Furthermore, a fraction of play bouts featured lying on the side while manipulating an object (5.5%), which has been referred to as self-handicap playing and is thought to have a signalling function in social play^47^, although only one study to date reports self-handicap during object play^48^.

Irrespective of the play type (social or non-social), we find that the duration of play bouts is significantly predicted by the type of object (Fig. 3). Individuals played with animal-derived or anthropogenic objects longer than with plant-derived or natural inorganic objects. Note that objects of the former two categories are not very common across the year: animal-derived objects (like small bones, fur or feathers) are only seasonally abundant and anthropogenic objects (like plastic bottles, baby pacifiers, FFP2 masks) are sometimes left by park visitors. In contrast, most objects of the two latter categories (plant-derived objects like leaves, sticks, bark; natural inorganic objects like stones, ice pieces) are available year-round. Hence, we may interpret our results as ravens exploring unfamiliar or novel items more thoroughly than familiar ones. These findings fit to the reports of juvenile ravens preferring novel objects^42^(own unpublished data) and experiments in captivity using unfamiliar objects to elicit play (e.g^11,13,14^). Longer interactions with unfamiliar or novel objects may allow individuals to reduce their neophobia towards anthropogenic objects in urban environments^49^, thus supporting the environment exploration hypothesis. Note, however, that out of the six play bouts recorded from adults in this data set, none used uncommon objects, but four involved play with plant-derived objects and two involved play with stones (Supplementary Table S3). Future studies may reveal if this pattern is stable and if (older) ravens simply choose objects for play according to their abundance in the environment (compare similar argument for bower birds^50^).

Taken together, our findings on free-ranging ravens validate various studies from captivity in respect to the occurrence across age class, context (social or non-social) and object characteristics of play, and highlight its dual function under field conditions. Further, we report the occurrence of object play in adult wild ravens and describe the variation in play patterns over seasons, thus emphasising the strength of studying play behaviours in wild populations. The decrease in object play with age class and the preference to play longer with relatively unfamiliar/novel objects suggest a key role of object play in exploring the physical environment, which goes well together with the ravens’ niche as feeding generalists and scavengers^51^. The high levels of play across (most) seasons, the prevalence of social and non-social object play in all age classes, and the report of specific object-directed behaviours (caching, self-handicapping) potentially encouraging social play indicate that object play may be also crucial for coping with a variable social environment and the uncertainties associated with the highly dynamic composition of raven groups^20,26,28^. Finally, we would like to emphasise the importance of long-term studies on corvid play behaviour in wild populations to understand the breadth of functions of play and its relevance in the species’ socio-ecological environment as demonstrated in studies on cetaceans^30^, primates^52^and birds^4,53^.

## Electronic supplementary material

Below is the link to the electronic supplementary material.

Supplementary Material 1

## Data Availability

The supplementary information has been submitted with this manuscript. The processed data sets and R code have been uploaded to an online repository and can be accessed here: https://doi.org/10.5281/zenodo.14185266.
