# Supplementary material for: Patterns of object play behaviour and its functional implications in free-flying common ravens
Source: Sci Rep. 2025 Jan 2;15:137. doi: 10.1038/s41598-024-83856-9 (PMC11697026; doi:10.1038/s41598-024-83856-9)
Supplement: Supplementary file 1 — Supplementary Material 1 [file 41598_2024_83856_MOESM1_ESM.pdf]

Patterns of object play behaviour and its functional implications in free-flying common ravens

Awani Bapat[1,2,*], Anna E. Kempf[1], Salome Friry[2,3], Palmyre H. Boucherie[1], Thomas Bugnyar[1,2,*]

[1]Department of Behavioral and Cognitive Biology, University of Vienna, Djerassiplatz 1, 1030 Vienna, Austria

[2]Konrad Lorenz Research Center for Behavior and Cognition, core facility of the University of Vienna, Fischerau 13, 4645 Grünau im Almtal, Austria

[3]Department of Evolutionary Biology and Environmental Studies, University of Zurich, Winterthurerstrasse 190, 8057 Zurich, Switzerland

*Corresponding authors: awani.bapat@univie.ac.at, thomas.bugnyar@univie.ac.at

Supplementary Information

Dataset 1 – Patterns of Occurrence of Object Play

Note S1

As part of the long-term focal observational sampling, we recorded 3257 focal observations on 163 individually marked birds (82 females, 81 males) between 2008-2015 and 2020-2022. Of these, 57 individuals were observed only for one year, 56 were observed over two years, 27 over three years, 7 over four years, and 6 over five years. Thus, 60 individuals were observed over more than one age class.

Table S1: Number of observations per individual and sex for each age class, and the percentage of individuals observed playing with objects at least once.

Age Class	Range of Focal observations per Individual	Number of Females	Number of Males	Total Number of Individuals	Percentage Individuals observed Playing
Juvenile	1-69	15	30	45	66.67%
Sub-adult Year 1	1-57	27	31	58	82.76%
Sub-adult Year 2	1-51	27	21	48	64.53%
Adult	1-128	43	39	82	51.22%

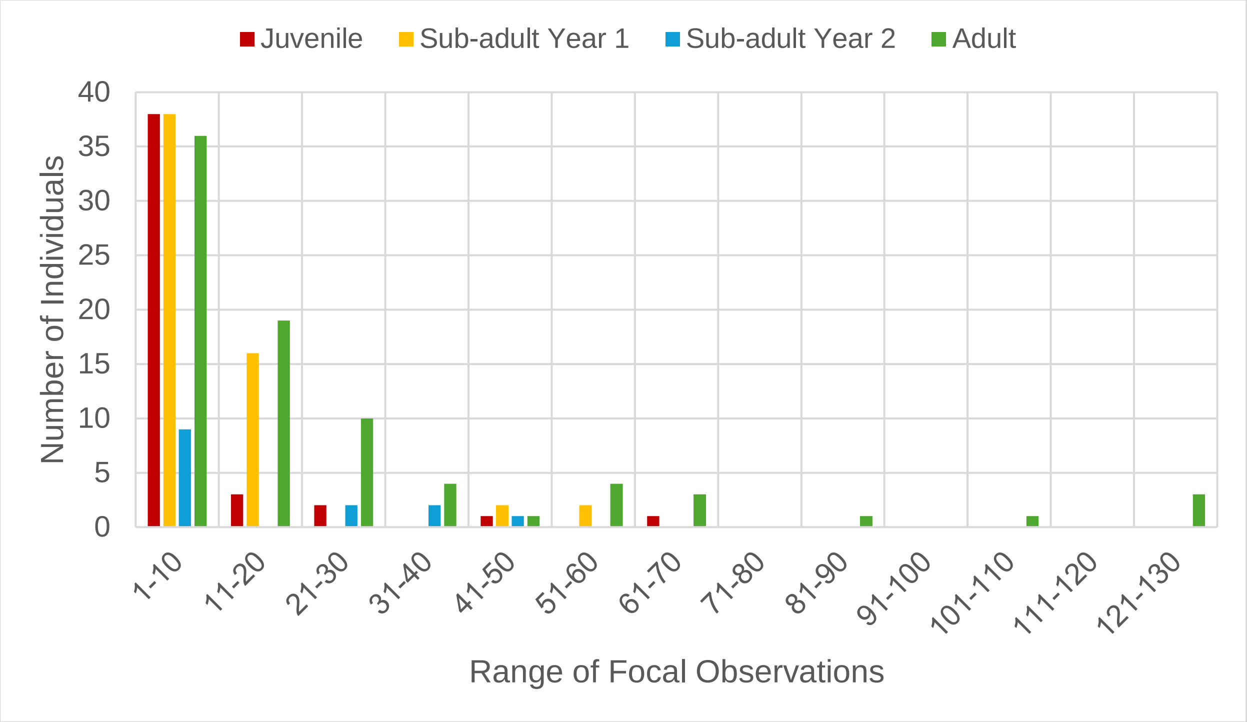

Figure S1: Range of focal observations for the individuals in the different age classes.

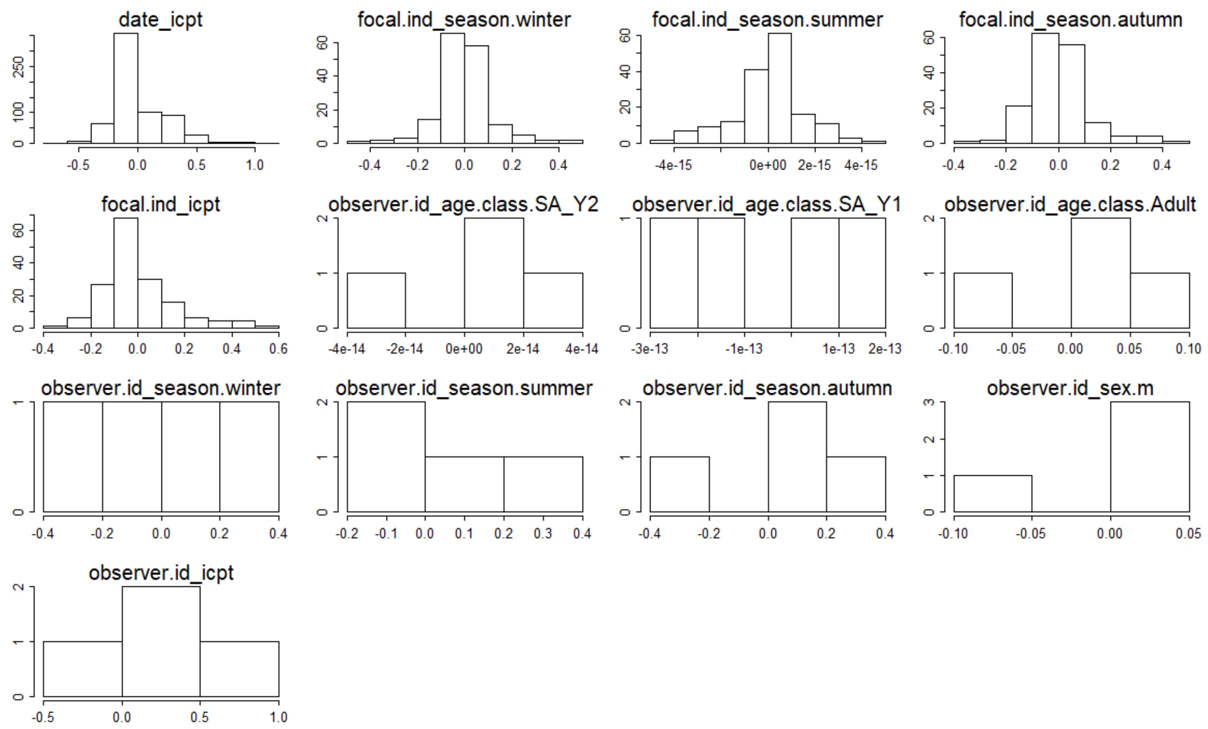

Figure S2: Distribution of best linear unbiased predictors (BLUPs) for model 1 (occurrence of object play). We visually inspected that the BLUPs were normally distributed with low within-subject variation (range of x-axis not exceeding ±3).

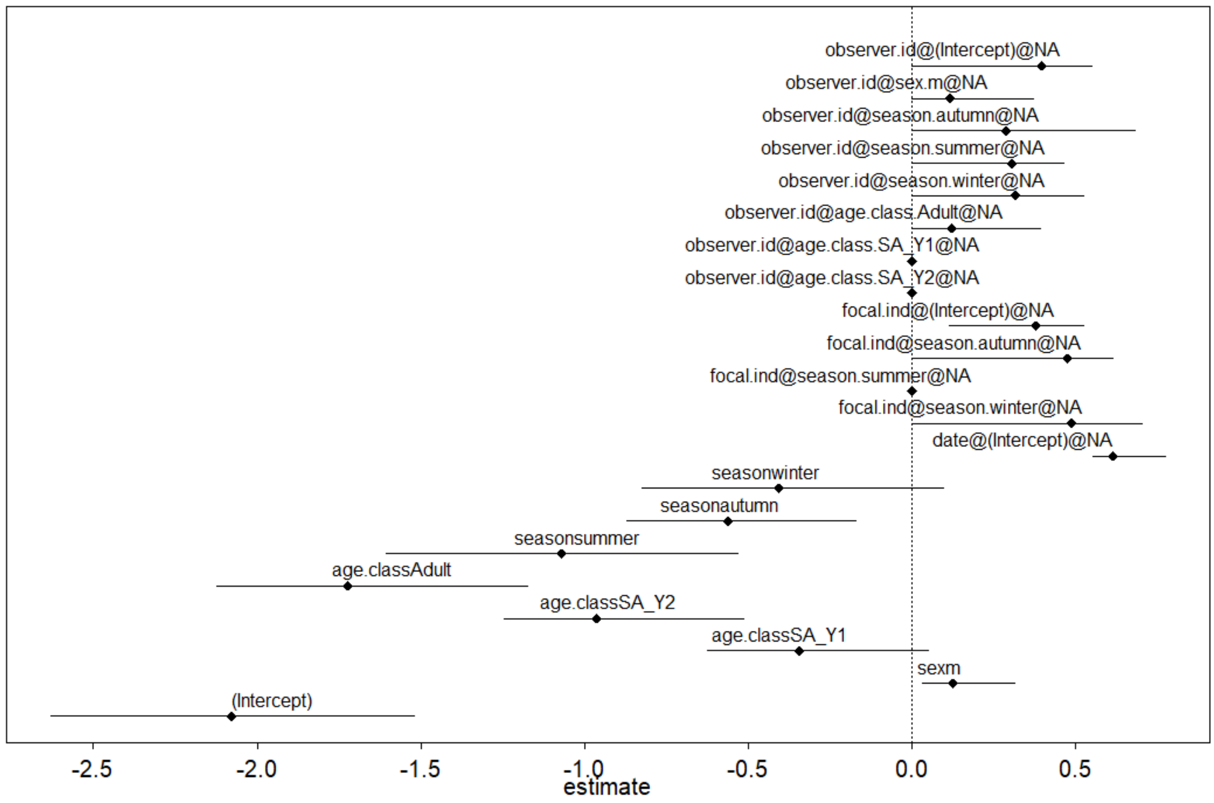

Figure S3: Model stability of estimates of model 1 (occurrence of object play). Points indicate the estimate of the full model and error bars indicate the range of estimates obtained after excluding each level of the random effects at a time.

Dataset 2 – Characteristics of Object Play

Note S2

To examine the characteristics of object play behaviour, 488 play bouts were recorded from 35 individually marked ravens (17 females, 18 males) between October 2021 – December 2023. Of these, 23 individuals were observed only for one year, 10 were observed over two years, and 2 over three years. Thus, 12 individuals were observed over more than one age class. Overall, we recorded observations from 23 juveniles, 13 sub-adults in their first year, 8 sub-adults in their second year, and 5 adults.

Table S2: List of object types observed during play observations recorded between October 2021-December 2023.

**Anthropogenic**	**Animal-derived**	**Plant-derived**	**Natural Inorganic**
baby pacifier	animal fur	bark	ice
coin	feather	dried plant	snow ball
cork	molusc shell	fircone	stone
FFP2 mask	small portable bones from carcass	leaf	
metal chain link		moss	
paper		nutshell	
plastic bottle/ bottle cap		straw	
plastic pieces		twig or branch	
plastic wrapper			
rubber ring			
sock, glove, other cloth pieces			
string			

Table S3: Frequency of types of objects played with by individuals of different age classes

Age Class	Type of Object				Total Play Bouts
	Anthropogenic	Animal-derived	Plant-derived	Natural Inorganic	
Juvenile	35	9	153	34	231
Sub-adult Year 1	27	18	111	34	190
Sub-adult Year 2	3	6	43	9	61
Adult	0	0	4	2	6
**Total**	**65**	**33**	**311**	**79**	**488**

Table S4: Number of social and non-social play bouts observed for each age class and sex.

		Number of Play Bouts		
		**Social**	**Non-Social**	**Total**
**Age class**	Juvenile	59	172	231
	Sub-adult Year 1	55	135	190
	Sub-adult Year 2	14	47	61
	Adult	3	3	6
**Sex**	Female	50	133	183
	Male	81	224	305

Table S5: Occurrence of different object play types, involving social interactions over objects or not.

**Type of Play**	Social Interactions over Objects	No Social Interactions
Manipulation	113	256
Manipulation + Locomotor	3	14
Manipulation + Caching	14	78
Manipulation + Caching + Locomotor	0	10

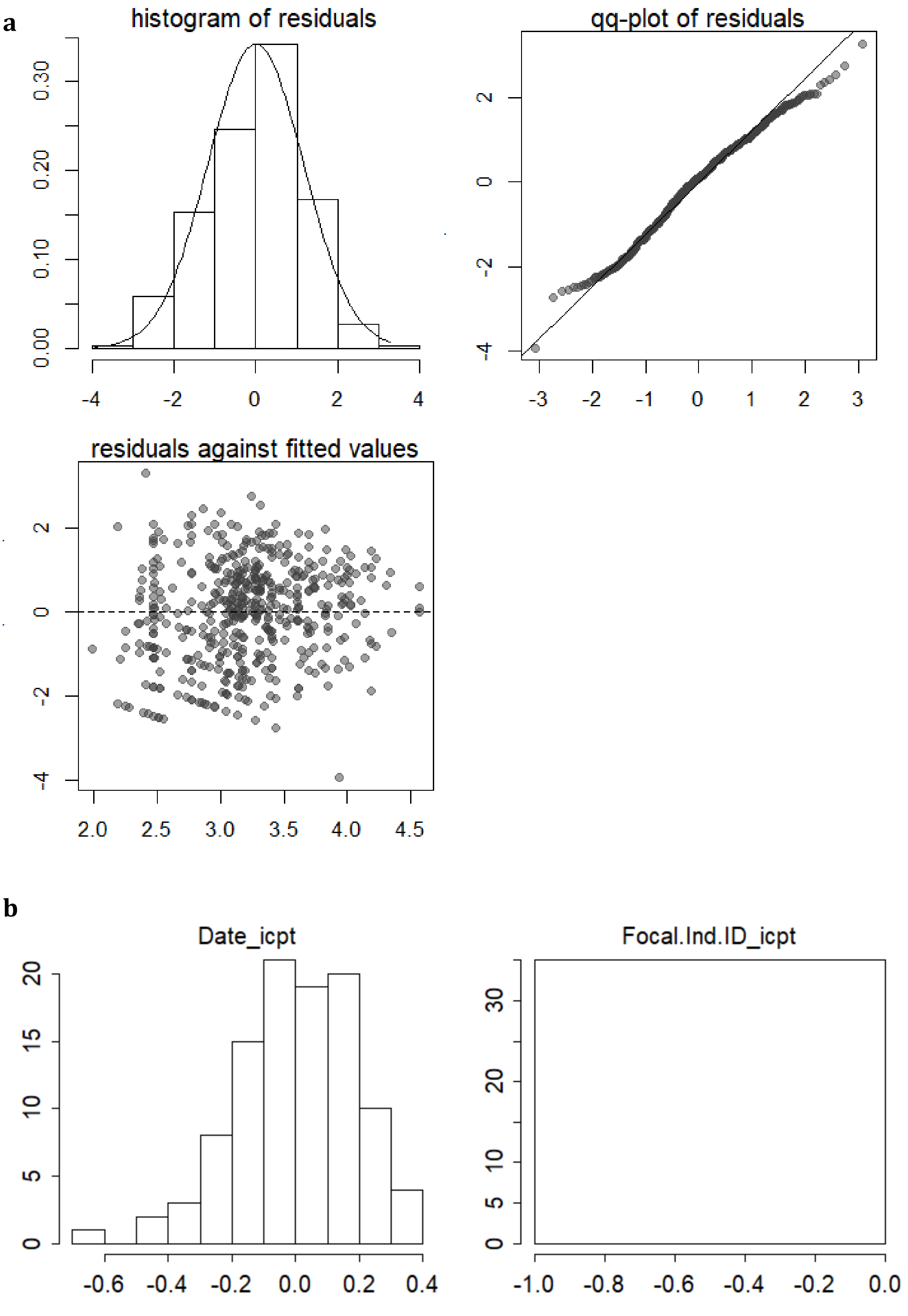

Figure S4: Model diagnostic plots for (a) normality and homogeneity of residuals and (b) BLUPs for model 2 (characteristics of object play). We visually inspected that the BLUPs were normally distributed with low within-subject variation (range of x-axis not exceeding ±3).

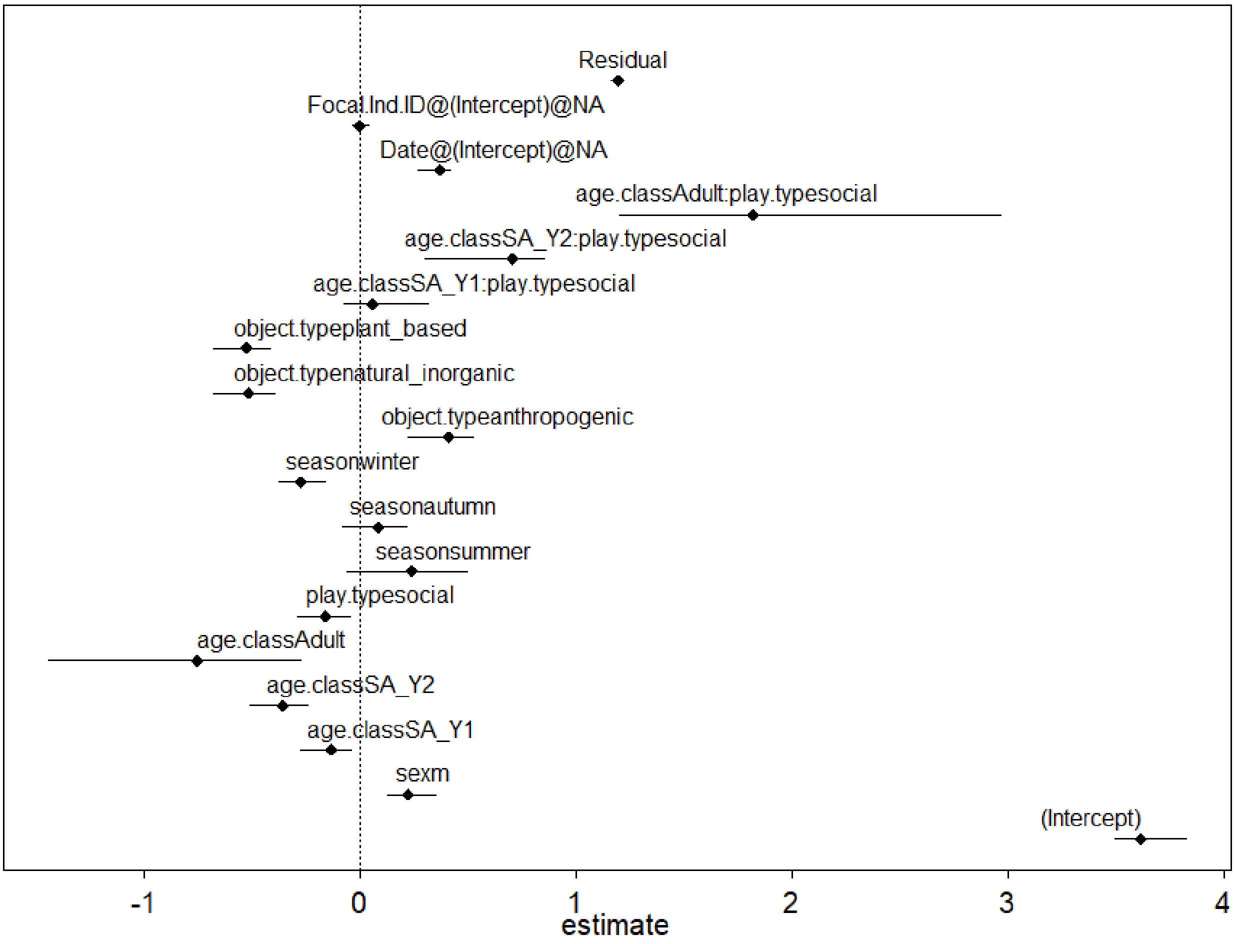

Figure S5: Model stability of estimates of model 2 (characteristics of object play). Points indicate the estimate of the full model and error bars indicate the range of estimates obtained after excluding each level of the random effects at a time. Note the large range of estimates for “age.classAdult” and “age.classAdult:play.typesocial”, indicating low stability of the model estimates corresponding to these predictors. This is likely due to the rare observations from adult individuals during the data collection period.

Table S6: Behavioural ethogram for play behaviours recorded.

Behaviour Type	Behaviour coded	Description
Frequency + Duration	Manipulate object	*Focal bird uses its beak and/or feet to manipulate, hold, or carry object*
Frequency + Duration	Co-manipulate object	*Focal bird manipulates object within reaching distance (0-50cm) of another bird manipulating the same or a different object*
Frequency + Duration	Cache or Retrieve object	*Focal bird carries object and/or sticks object into crevice and/or covers object with soil and substrate, or retrieves object from crevice or covered substrate*
Frequency	Transfer object	*Focal bird transfers an object to another bird*
Frequency	Show/offer object	*Focal bird visually presents an object to another bird, typically by holding it in tip of its beak and bending over and/or holding the beak and object pointed towards or in front of the other's beak*
Frequency	Request object	*Focal bird gently touches another bird's beak and/or the object in the other's possession.*
Frequency	Steal object	*Focal bird approaches another bird handling an object and tries to take the object by force*
Frequency	Pilfer object	*Focal bird approaches another bird while caching and tries to take the object out of the cache / searches at another bird's cache when the cacher is absent and tries to find the item*
Frequency + Duration	Hanging upside down	*Focal bird hangs upside down by its feet and/or its beak*
Frequency + Duration	Sliding on belly	*Focal bird lies down on its belly and moves its feet, or lies on its back and moves its feet, or turns around its body axes in a rolling fashion*
Frequency + Duration	Play jump or play fight	*Focal bird jumps in the air and turns around its axes and/or jumps/flies at another bird in an exaggerated way, typically by giving characteristic calls*
